# Prussian Blue–based microsensors in short-circuit mode: a simplified approach for localized hydrogen peroxide detection

**DOI:** 10.1007/s00216-026-06586-z

**Published:** 2026-06-09

**Authors:** Elena Daboss, Sven Daboss, Manfred Frick, Christine Kranz

**Affiliations:** 1 Institute of Analytical and Bioanalytical Chemistry, Ulm University, Albert-Einstein-Allee 11, 89081 Ulm, Germany; 2 Institute of General Physiology, Ulm University, Albert-Einstein Allee 11, 89081 Ulm, Germany

**Keywords:** Prussian Blue–based microsensors, Hydrogen peroxide detection, Scanning electrochemical microscopy (SECM), Short-circuit mode, ATP microbiosensor

## Abstract

We report on a Prussian Blue (PB)–based microsensor with an integrated silver quasi-reference electrode (QRE) for selective hydrogen peroxide (H_2_O_2_) reduction using the excellent electrocatalytic properties of PB in a so-called short-circuit mode. This “single-electrode” configuration eliminates the need for a potentiostat allowing a simplified read-out. The microsensors are either based on PB or on PB-nickel hexacyanoferrate (PB-NiHCF) and demonstrate high sensitivity (1.8 A M^−1^ cm^−2^ and 0.32 A M^−1^ cm^−2^ for PB-based and PB-NiHCF-based microsensors, respectively), operational stability (maintaining performance for 4 h under harsh conditions in 1 mM H_2_O_2_), and selectivity toward H_2_O_2_ with negligible interference from common reductants such as ascorbic acid and catecholamines. By incorporating a platinum-black (Pt-B) sublayer, the PB film deposition could be significantly improved, yielding a threefold sensitivity increase and a 14-fold enhancement in operational stability. The microsensors were successfully applied as probes for scanning electrochemical microscopy (SECM) measurements to map H_2_O_2_ evolution. Furthermore, by modification of the PB-modified Pt microelectrode (ME) with glucose oxidase (GOx) and hexokinase (HEX), an adenosine triphosphate (ATP) microbiosensor was developed and applied for extracellular ATP release detection. The ATP determination relies on the competitive GOx- and HEX-catalyzed reactions for glucose. These results highlight the potential of PB-based MEs in short-circuit mode for localized electrochemical studies of not only catalytic systems where monitoring H_2_O_2_ concentration is of primary interest, but also various biological systems, such as living cells, where H_2_O_2_ is generated as a byproduct of enzymatic reactions.

**Graphical abstract:**

**Figure:**
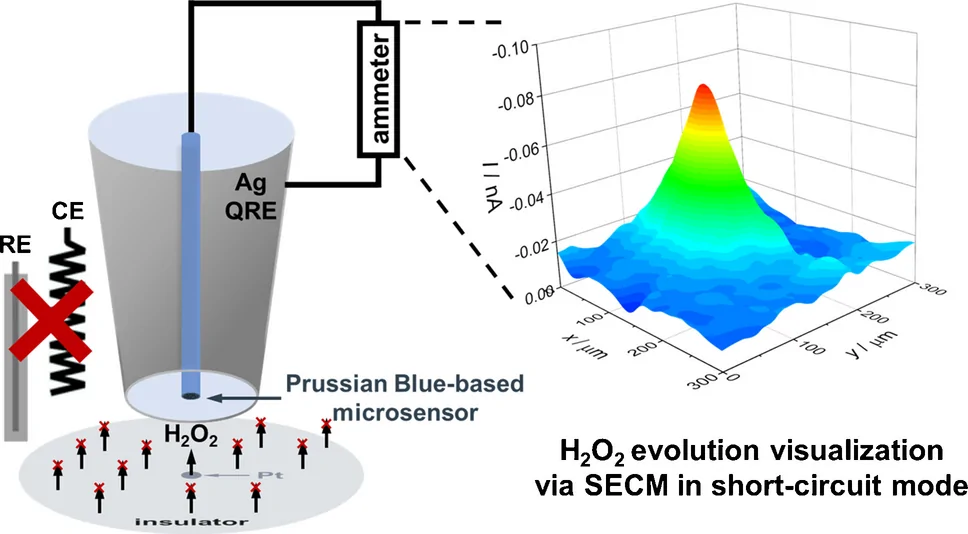

**Supplementary Information:**

The online version contains supplementary material available at https://doi.org/10.1007/s00216-026-06586-z.

## Introduction

Hydrogen peroxide (H_2_O_2_) belongs to the 100 most important chemical compounds and is a key analyte with importance in life sciences as well as industrial applications, for example, as an oxidizing agent in production processes [1] or as a disinfection agent in the health sector [2]. In addition, H_2_O_2_ has gained importance as green liquid fuel, which can be produced by light-driven alcohol oxidation [3]. In living cells, H_2_O_2_ functions as an intracellular signaling molecule regulating kinase-driven pathways [4], and as a crucial reactive oxygen species (ROS) in oxidative stress and immune responses [5]. In biotechnology, it is a major product of oxidase-based enzymatic reactions (e.g., glucose oxidase (GOx)), and thus serves as the basis for the most widely used biosensors [6].

While conventional analytical measurements provide bulk concentrations, local quantification of H_2_O_2_ is often required, e.g., for biomedically relevant samples such as cell studies, where H_2_O_2_ is produced at micrometer-scale domains [7, 8]. In addition, analyte concentrations (either H_2_O_2_ itself or other target species that are converted to H_2_O_2_) in the bulk solution are in numerous cases below the detection limits of conventional methods [9–11]. Scanning electrochemical microscopy (SECM) represents a powerful electroanalytical technique which provides real-time localized information on electrocatalytic activity and the spatial distribution of redox-active species [12].

Various SECM-based local H_2_O_2_ measurements have been reported using platinum (Pt) [13–18] and Pt-B [19–21] microelectrodes (MEs). The high anodic operation potential (0.6–0.7 V for Pt-MEs and 0.3 V vs. Ag/AgCl (hereafter, all potentials are reported vs. Ag/AgCl reference electrode, unless otherwise specified) for Pt-B microsensors, respectively) makes them prone to interference from easily oxidized species such as ascorbate or dopamine [22]. Alternatively, peroxidase-based microbiosensors have been used [23], which provide enzymatic selectivity but suffer from limited stability of the enzyme. PB, often referred to as an “artificial peroxidase,” catalyzes H_2_O_2_ reduction at potentials close to 0 V, thus minimizing interference from common reductants [24, 25]. For example, PB-modified MEs have been used in substrate-generation/tip-collection (SG/TC) mode, to map locally generated H_2_O_2_ [26, 27].

A limitation of PB is its low stability under prolonged operation times, particularly in neutral or alkaline media. The electrochemical reduction of H_2_O_2_ under such conditions generates hydroxide ions, which are able to solubilize PB [28]. To overcome this disadvantage, PB has been stabilized by incorporation of transition metals, e.g., with nickel hexacyanoferrate (NiHCF) among the most effective matrixes [26, 29, 30]. Such PB-NiHCF hybrid films combine high selectivity with improved robustness, enabling longer-term H_2_O_2_ monitoring (4 h continuous monitoring in 1 mM H_2_O_2_ solution) using PB-NiHCF-modified MEs [26]. Recently, the concept of operating PB-based sensors in short-circuit mode has been introduced [31]. In this configuration, a current generated by a short-circuiting of the PB-modified screen-printed electrode with an Ag QRE using an ammeter (without an externally applied potential) is proportional to the bulk H_2_O_2_ concentration. This approach has so far been only demonstrated for macroelectrodes and has to our best knowledge not yet been adapted to SECM-compatible microsensor for local H_2_O_2_ evolution determination and imaging.

Here, we present highly sensitive, selective, and stable PB-based H_2_O_2_ microsensors, integrated with an Ag QRE into a single microprobe, enabling a simplified short-circuit operation. The developed microsensors were further applied as SECM probes for spatially resolved visualization of H_2_O_2_ production in proof-of-principle measurements at a biased microelectrode. Moreover, ATP microbiosensors were constructed and employed for real-time monitoring of extracellular ATP release, highlighting the versatility of the platform for both catalytic and bioanalytical applications.

## Materials and methods

### Chemicals and materials

All solutions were prepared with ultrapure water (Elga water system, UK, conductivity 18.0 MΩ·cm). Potassium dihydrogen phosphate (KH_2_PO_4_), dipotassium hydrogen orthophosphate (K_2_HPO_4_), disodium hydrogen orthophosphate (Na_2_HPO_4_), potassium chloride (KCl), sodium chloride (NaCl), calcium chloride (CaCl_2_), magnesium chloride (MgCl_2_), sodium hydroxide (NaOH), bisphenol A (BA), tetraethylenepentamine (TEPA), formaldehyde, phorbol 12-myristate 13-acetate (PMA), hydrogen hexachloroplatinate (H_2_[PtCl_6_]), lead(II) nitrate (Pb(NO_3_)_2_), tin(II) chloride (SnCl_2_), glucose monohydrate, potassium hexacyanoferrate(III) (K_3_[Fe(CN)_6_]), iron(III) chloride hexahydrate (FeCl_3_·6H_2_O), nickel(II) chloride (NiCl_2_), l-ascorbic acid, norepinephrine, dopamine hydrochloride, silver nitrate (AgNO_3_, 1 M solution), hydrogen peroxide (30% solution), GOx from *Aspergillus niger* type X-S (148 U mg^−1^), ammonia (28% solution), all organic solvents, hydrochloric acid (35% solution), nitric acid (70% solution), and sulfuric acid (85% solution) were obtained from Merck, Germany. Hexokinase (HEX) from yeast (209 U mg^−1^) was purchased from Calbiochem (Merck, Germany). Hexaammineruthenium(III) chloride ([Ru(NH_3_)_6_]Cl_3_) was purchased from Acros Organics, Belgium. Pt microwires were purchased from Goodfellow, UK; glass capillaries were obtained from Hilgenberg, Germany. Polishing materials were obtained from Buehler ITW Test & Measurement GmbH, Germany, and Allied High-Tech Products Inc., USA, respectively.

### Instrumentation

Electrochemical measurements in a three-electrode setup were conducted in a cell with an Ag|AgCl|KCl (sat.) reference electrode and a Pt counter electrode using a CHI660C potentiostat (CH Instruments, USA) or a Palmsens 4 potentiostat (PalmSens BV, The Netherlands) in combination with a Faraday cage. For the measurements in short-circuit mode, a Palmsens 4 potentiostat in Zero Resistance Ammeter (ZRA) mode was used. Scanning electron microscopy (SEM) imaging was performed using a Quanta 3D FEG microscope (Thermo Fisher Scientific, USA) and energy-dispersive X-ray (EDX) mapping was performed with EDAX, SDD Apollo XV (EDAX, USA). The SECM measurements were performed in constant-height mode at a distance of 20 µm using a custom-built SECM setup (a micropositioning system (Scientific Precision Instruments, Germany) in combination with a sample stage, located in a Faraday cage and controlled via the SECMx software (G. Wittstock, Carl von Ossietzky University of Oldenburg, Germany)). Data evaluation was done using the MIRA software package, version 8.1 (G. Wittstock, Carl von Ossietzky University of Oldenburg, Germany). For the epithelial cell studies, a positioning system (z-stepper motor equipped with the K-Cube® Stepper Motor Controller (Thorlabs, Germany)) was mounted on the stage of an Olympus IX81 inverted fluorescence microscope (Olympus, Japan).

### Methods

#### Microelectrode fabrication

MEs were fabricated as described elsewhere [32] by sealing a Pt wire (Ø = 25 µm) under vacuum in borosilicate glass followed by consecutive grinding and polishing steps and finally cleaning in an ultrasonic bath for 15 min in ultrapure water. Silver (Ag) QREs were formed onto a glass capillary which surrounds the Pt wire [33]. First, the surface was sensitized with tin(II) chloride, and then a silver-diammine complex (Tollens’ reagent) was applied in the presence of glucose, which reduces Ag⁺ to metallic silver, forming a reflective coating.

#### PB and PB-NiHCF deposition

The electrodeposition of PB was carried out on Pt-MEs (both bare and Pt-B-modified) using cyclic voltammetry (CV) in the range from 0.4 to 0.75 V at a scan rate of 0.02 V·s^−1^ [27]. The solution contained 1.25 mM K_3_[Fe(CN)_6_], 1.25 mM FeCl_3_, and 0.1 M KCl in 0.1 M HCl. For the deposition of NiHCF, a solution containing 0.5 mM K_3_[Fe(CN)_6_], 1 mM NiCl_2_, and 0.5 M KCl in 0.1 M HCl was used. The deposition was performed by cycling the applied potential from 0.0 to 0.75 V at a scan rate of 0.1 V·s^−1^. Layer-by-layer deposition of PB-NiHCF was done as described elsewhere [26]. A PB layer was deposited onto Pt-MEs by CV (2 cycles), followed by subsequent deposition of NiHCF by CV (2 cycles). This procedure was repeated three times, with the electrodes rinsed with ultrapure water after each step. Then the deposited films were annealed at 80 °C for 1 h.

#### Pt-B deposition

Pt-B was electrochemically deposited onto Pt-MEs according to a procedure adapted from Ben-Amor et al. [20] by applying a constant potential of −0.06 V for 40 s in a solution of 31 mM H_2_PtCl_6_ and 0.67 mM Pb(NO_3_)_2_ in phosphate buffer saline (PBS).

#### ATP biosensor fabrication

First, PB was electrodeposited on a Pt-ME and then annealed at 80 °C for 1 h. The number of PB deposition cycles was varied from 1 to 15. A poly(benzoxazine) layer was used as an immobilization matrix for GOx and HEX onto PB-modified Pt-MEs. The electropolymerization was carried out in a mixture of bisphenol A-tetraethylenepentaamine (BA-TEPA) [34] and GOx/HEX in a ratio of 1:2.5 w/w, with a 1:1 activity ratio for the two enzymes [35]. A potential pulse sequence of 1.7 V/0.05 s, 0.0 V/0.5 s was used [35]; the total number of repetitions was varied from 2 to 20.

#### Cell isolation and cell culture

ATII cells were isolated from lung tissue according to the procedure of Dobbs et al. for isolation of ATII cells from rat lung [36] with minor modifications as recently described [37]. Lungs were harvested from male Sprague-Dawley rats (Charles River, Germany) in accordance with a protocol reviewed and approved by the Regierungspräsidium Tübingen. Following the isolation, ATII cells were collected in MucilAir™ Medium (Epithelix Sàrl, Geneva, Switzerland) with 25.6 µg·mL^-1^ gentamicin (Thermo Fisher Scientific, Germany) and cultured for 24 h for cell adhesion.

#### Statistics

All experiments were repeated at least three times using three different sensors, and the data are represented by mean value ± standard deviation (SD).

## Results and discussion

### Prussian Blue–based microsensor for H_2_O_2_ detection in short-circuit mode

**Fig. 1:**
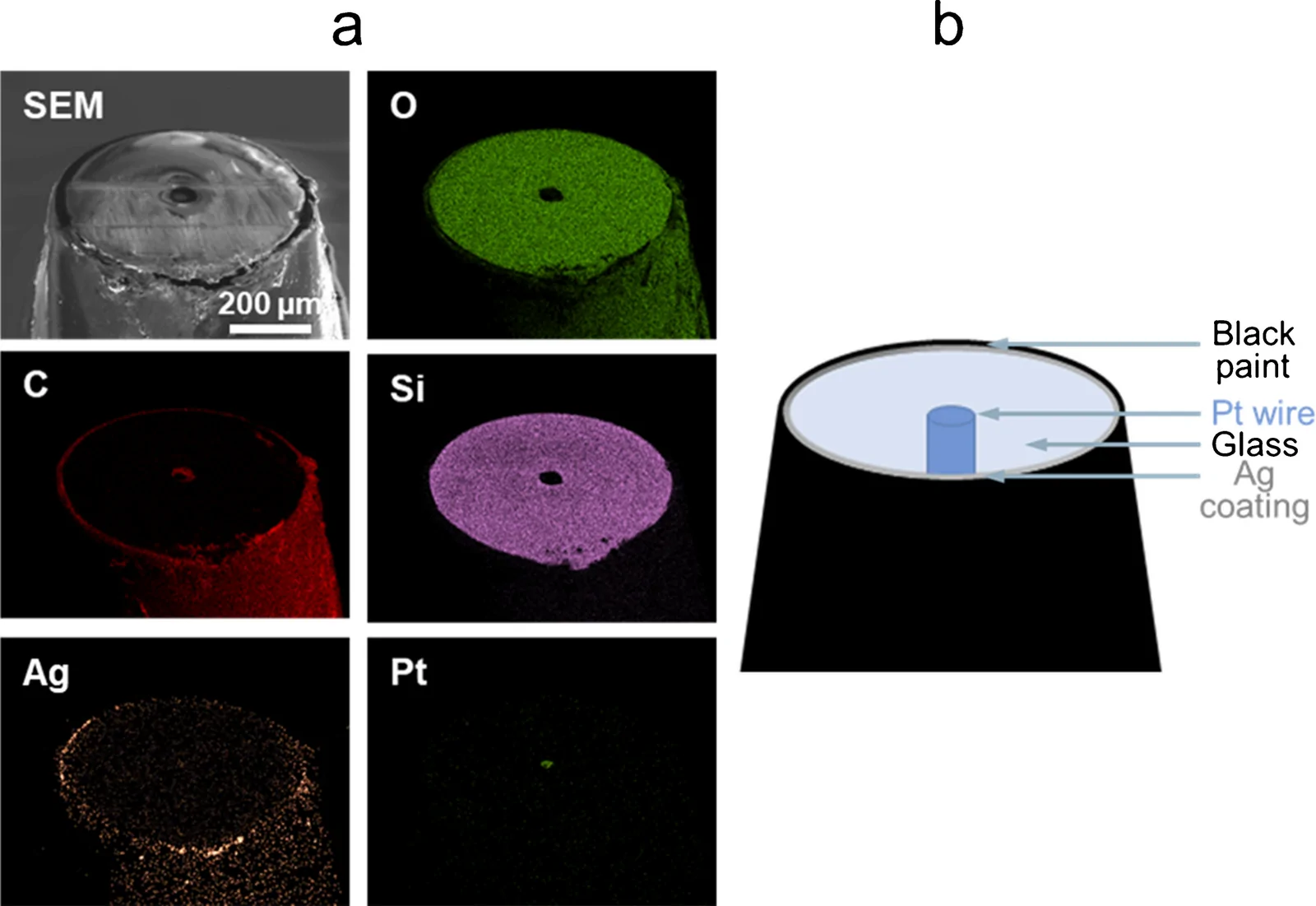
**a** SEM image and corresponding O, C, Si, Ag, and Pt EDX maps of a Pt-ME modified with an integrated silver layer as QRE (the scale bar is 200 µm) and **b** a scheme of the combined electrode

First, the modified Pt-MEs for the short-circuiting were fabricated. By integrating the working ME and the QRE, the setup could be simplified using a single modified ME for measurements. For this purpose, an Ag layer was deposited [33] onto a glass sheath which surrounds the Pt wire. The SEM and EDX maps shown in Fig. 1a reveal the silver coating. To protect the silver layer from the mechanical damage, it was covered with the black paint before the electrochemical measurements. To restore a clean, exposed electroactive area, the MEs were gently repolished. Figure 1b shows the scheme of the obtained combined electrode.

The PB layer was electrodeposited onto the Pt micro-disc of the fabricated electrodes. Cyclic voltammograms of the PB-modified ME vs. the deposited Ag electrode were compared vs. an external Ag/AgCl reference electrode (Fig. S1). The observed potential shift is around 20 mV, which is acceptable for using the silver QRE.

**Fig. 2:**
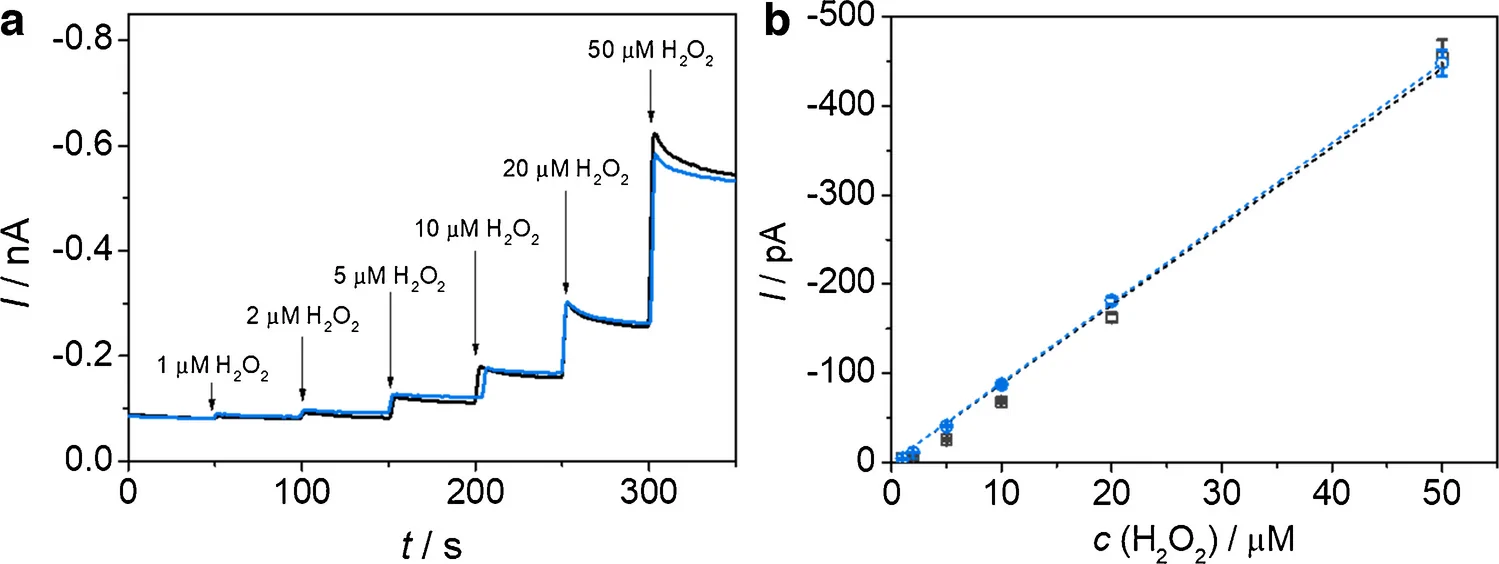
**a** Current responses of the H_2_O_2_ microsensor based on PB using the conventional three-electrode setup powered by a potentiostat, −0.05 V (gray line) and in short-circuit mode (blue line), in 50 mM K_2_HPO_4_/KH_2_PO_4_, pH 6.0, with 0.1 M KCl. **b** Corresponding calibration graphs, 3 different microsensors for each mode (*n* = 3)

Current responses and corresponding calibration graphs for the PB-based microsensor tested in a conventional three-electrode setup at −0.05 V (gray curve and points) and in short-circuit mode (blue curve and points) are shown in Fig. 2. As expected, the sensors exhibit comparable analytical performance in terms of sensitivity (1.8 ± 0.1 A M^−1^ cm^−2^), response time (less than 10 s), and a linear range (1–100 µM H_2_O_2_).

The measurement of H_2_O_2_ using Pt electrodes or metal-based electrocatalysts (e.g., Pt-B) is often influenced by electroactive reductants, which are co-oxidized at similar anodic potentials [6]. On the contrary, electrochemical reduction of H_2_O_2_ using PB-modified electrodes leads to significantly reduced interfering issues. Therefore, the selectivity of the bare Pt, the Pt-B-based, and the PB-based microsensors (in short-circuit mode) was evaluated against several electroactive compounds such as ascorbate, norepinephrine, and dopamine, chosen as representative reducing interferents commonly present in biological environments. In this case, only the microsensor based on PB is selective to H_2_O_2_ (Fig. S2). A cathodic current for H_2_O_2_ reduction is observed, whereas the interferent species (e.g., ascorbate) are oxidized leading to an anodic current response. A 10-fold excess of ascorbate or norepinephrine and a 70-fold excess of dopamine are required to produce a sensor response comparable to that obtained for micromolar concentrations of H_2_O_2_. In contrast, bare Pt-MEs exhibit a higher response toward interfering components than toward H_2_O_2_ (*k* = *I* (H_2_O_2_)/*I* (interfering compounds) << 1). Pt-B-modified Pt-MEs produce signals of comparable magnitude for the same concentrations of H_2_O_2_ and other reductants (*k* = 1 − 2). In addition, the limits of detection (LOD) for Pt-B (287 ± 14 nM) and PB-based (191 ± 10 nM) microsensors are, respectively, 10 and 15 times lower than the LOD for the non-modified Pt microsensor (2.9 ± 0.2 µM).

**Fig. 3:**
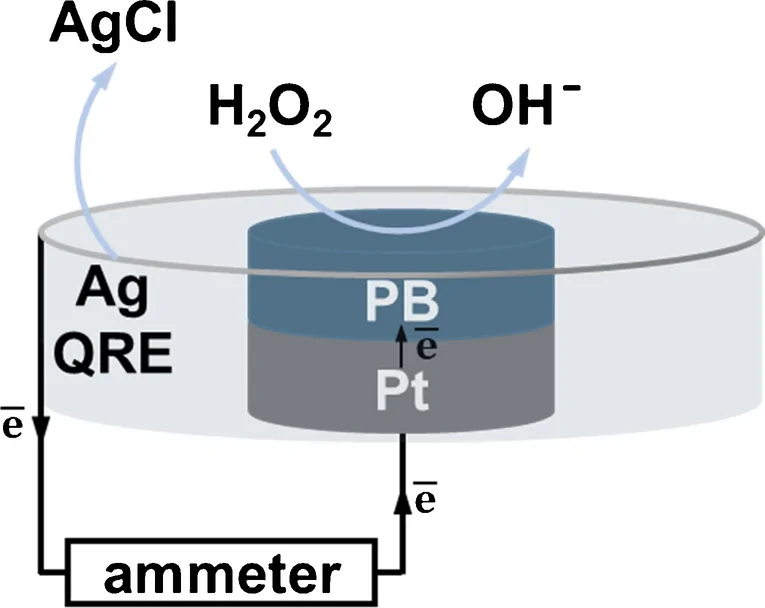
The scheme of the H_2_O_2_ microsensor operation in short-circuit mode

It was shown that under short-circuit conditions, the electrode potential is fixed near that of the silver QRE, keeping PB at its optimal working potential for H_2_O_2_ reduction [31]. In the absence of an external power supply, the system operates as a galvanic cell in which electrons required for the catalytic cycle are provided by the anodic dissolution of silver (Eq. 1). These electrons reduce PB to Prussian White (Eq. 2), which subsequently reduces H_2_O_2_ (Eq. 3), regenerating the catalyst and sustaining a continuous faradaic current proportional to the H_2_O_2_ concentration. The scheme of the fabricated microsensor operation is demonstrated in Fig. 3.

1 $$\mathrm{A}\mathrm{g}+ {\mathrm{C}\mathrm{l}}^{-} \to \mathrm{A}\mathrm{g}\mathrm{C}\mathrm{l}+ {\mathrm{e}^{-}}$$

2 $$\mathrm{K}{\mathrm{F}\mathrm{e}}^{\mathrm{I}\mathrm{I}\mathrm{I}}\left[{\mathrm{F}\mathrm{e}}^{\mathrm{I}\mathrm{I}}{\left(\mathrm{C}\mathrm{N}\right)}_{6}\right]+ {\mathrm{e}^{-} }+{\mathrm{K}}^{+}\leftrightarrow {\mathrm{K}}_{2}{\mathrm{F}\mathrm{e}}^{\mathrm{I}\mathrm{I}}\left[{\mathrm{F}\mathrm{e}}^{\mathrm{I}\mathrm{I}}{\left(\mathrm{C}\mathrm{N}\right)}_{6}\right]$$

3 $${2\mathrm{K}}_{2}{\mathrm{F}\mathrm{e}}^{\mathrm{I}\mathrm{I}}\left[{\mathrm{F}\mathrm{e}}^{\mathrm{I}\mathrm{I}}{\left(\mathrm{C}\mathrm{N}\right)}_{6}\right]+{\mathrm{H}}_{2}{\mathrm{O}}_{2}\to 2\mathrm{K}{\mathrm{F}\mathrm{e}}^{\mathrm{I}\mathrm{I}\mathrm{I}}\left[{\mathrm{F}\mathrm{e}}^{\mathrm{I}\mathrm{I}}{\left(\mathrm{C}\mathrm{N}\right)}_{6}\right]+2{\mathrm{O}\mathrm{H}}^{-}+2{\mathrm{K}}^{+}$$

**Fig. 4:**
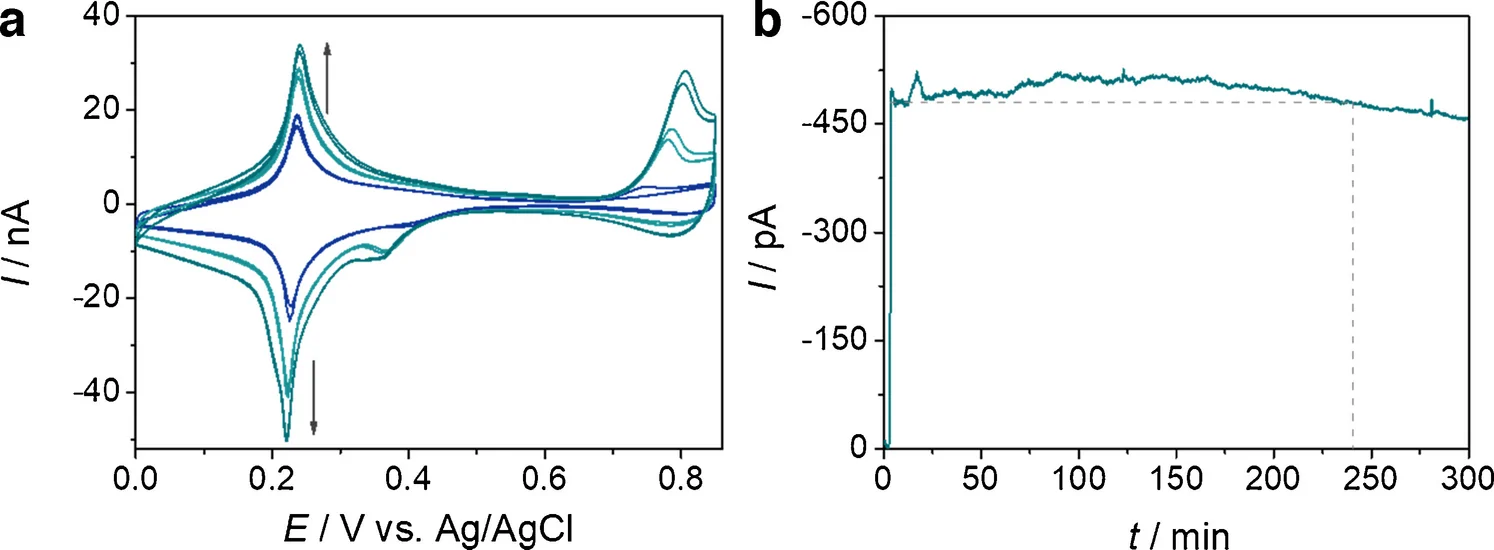
**a** Layer-by-layer deposition of NiHCF onto PB; 0.1 M HCl and 0.5 M KCl, scan rate 0.1 V s^−1^ and **b** operational stability of H_2_O_2_ sensor based on PB-NiHCF in short-circuit mode; 1 mM H_2_O_2_ in 50 mM PBS, pH 6.0, with 0.1 M KCl. Dashed lines indicate the period when current remains ≥ its initial value

An important characteristic of the reference electrode in the used short-circuit mode is its stability in monitoring of high H_2_O_2_ concentrations. Due to their inherent instability in neutral and alkaline media, which limits the applicability of PB-based sensors for long-term continuous monitoring [28], stabilized H_2_O_2_ microsensors based on PB-NiHCF were fabricated according to [26] (Fig. 4a) and then studied under harsh conditions of 1 mM H_2_O_2_ to prove sufficient QRE stability. No decay of the current response was observed for 4 h (Fig. 4b). This is consistent with literature data obtained under similar conditions using an external Ag/AgCl reference electrode in a three-electrode setup [26]. Thus, we can demonstrate that the Ag QRE surrounding the glass sheath of the microsensor is not limiting the stability of the H_2_O_2_ microsensor.

In addition, the stability of the Ag QRE potential was investigated. The microsensors were stored for 2 weeks in air and in PBS with pH 7.4, respectively. The potential remains almost unchanged and only minor fluctuations were observed (Fig. S3). Accordingly, the QRE does not require any special storage conditions and does not alter the potential under the tested conditions for 14 days.

The achieved analytical performance—particularly the high operational stability of both the electrocatalyst-modified ME and the QRE—enables using such a sensor for long-term measurements, e.g., for SECM mapping.

### High-stability H_2_O_2_ microsensors

To improve the performance of the PB microsensors, Pt-MEs were firstly modified with a Pt-B film (Fig. S4a). The recorded cyclic voltammogram in 10 mM [Ru(NH_3_)_6_]Cl_3_/0.1 M KCl solution after Pt-B deposition demonstrates the increase in electroactive surface area (EASA is 0.0013 ± 0.0001 cm^2^, evaluated from Fig. S5) resulting in a significant increase in the current response for the modified ME (Fig. S4b). As previously shown, the Pt-B deposition on MEs results in a highly porous structure with channels and gorges extending throughout the Pt-B volume, which is visible by the increase in capacitive current as shown in Fig. S4b [19, 38].

**Fig. 5:**
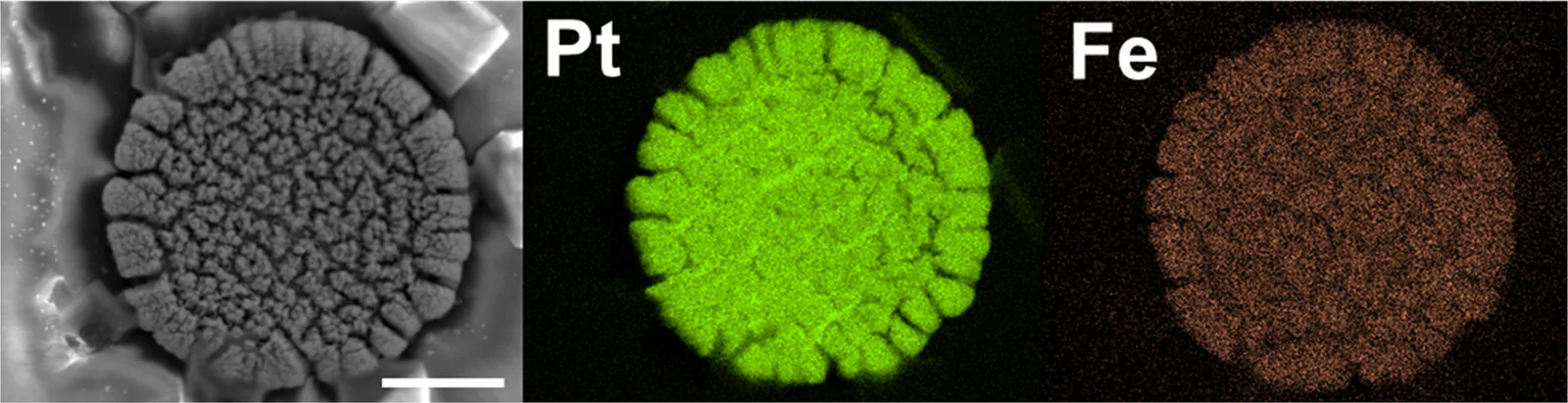
SEM image (left) and corresponding EDX elemental maps of Pt and Fe for a Pt-B/Pt-ME after PB deposition. Scale bar is 10 µm

PB was then electrodeposited via CV onto the Pt-B-modified ME (Fig. S4c). The EDX maps show that the iron (Fe) signal closely follows the porous morphology of the Pt-B layer, confirming that the PB film forms a homogeneous coverage (Fig. 5). The cyclic voltammograms recorded before and after three consecutive calibrations (Fig. S4d, blue and teal curves, respectively), characterized by sharp, symmetric redox peaks and a narrow peak separation (~ 10 mV), indicate that the PB film possesses a uniform and regular structure with consistent electrochemical activity across the electrode surface [39].

**Fig. 6:**
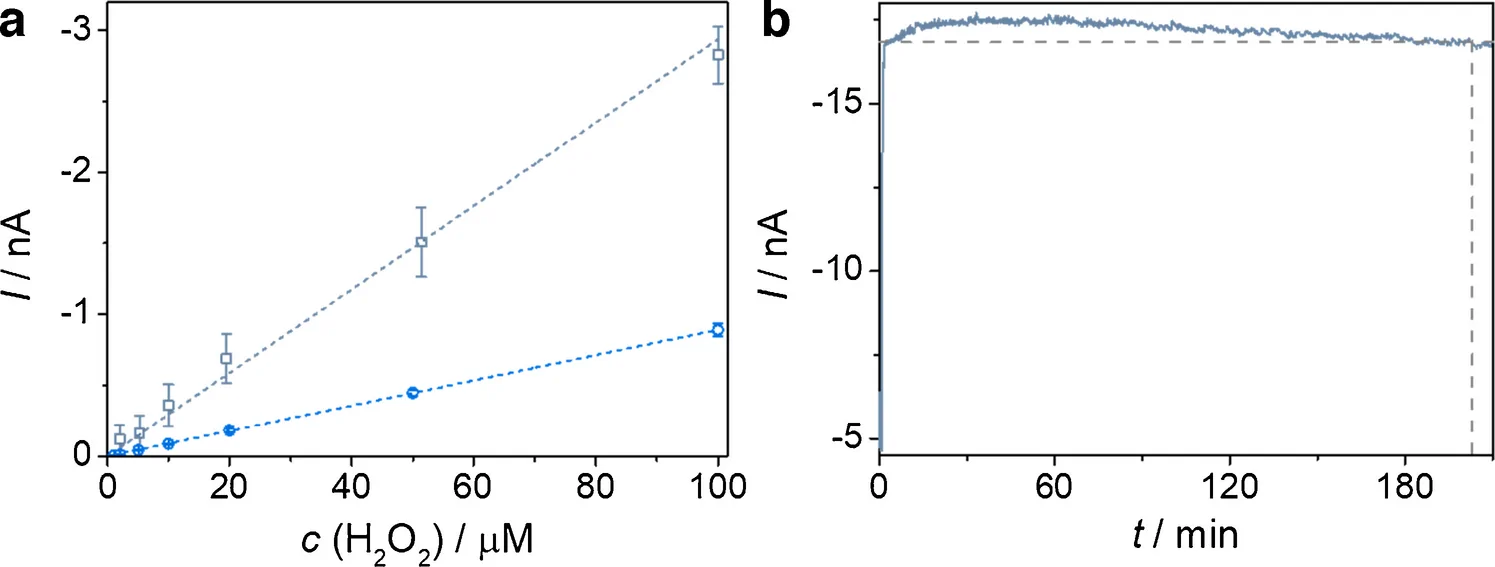
**a** Calibration graphs for the sensors based on PB/Pt-B (square) and PB (circle) (*n* = 3, three different microsensors). **b** Operational stability of the sensor based on PB/Pt-B in 1 mM H_2_O_2_; short-circuit mode, 50 mM PBS, pH 6.0, with 0.1 M KCl. Dashed lines indicate the period when current remains ≥ its initial value

The obtained PB/Pt-B sensor was tested for H_2_O_2_ measurements by recording the current response in short-circuit mode (Fig. S6). The corresponding calibration curve was compared with the PB-modified electrode without the Pt‑B underlayer (Fig. 6a). For both sensors, the current response increases linearly with H_2_O_2_ concentration in the 1–100 µM range; however, the PB/Pt‑B microsensor exhibits a markedly higher slope, reflecting a three times enhancement in sensitivity (29 ± 2 µA M^−1^ vs. 8.9 ± 0.4 µA M^−1^). The improved performance is attributed to the high electroactive surface area and favorable morphology of the Pt‑B layer, which promotes efficient PB deposition. The cyclic voltammograms recorded before and after three consecutive calibrations (Fig. S4d) demonstrate that the electrocatalytic film retains its redox activity and structure (the redox peaks became marginally sharper and the peak current slightly increased), confirming its operational stability. Furthermore, the microsensor does not display any decay in current response during more than 3 h (Fig. 6b), what is sufficient regarding the typical duration of a SECM imaging experiment.

Table 1 summarizes the key characteristics of the PB-based and PB-NiHCF-based microsensors presented in this work.

**Table 1:** Analytical performances and operational stability of the H_2_O_2_ microsensors; 50 mM PBS, pH 6.0, with 0.1 M KCl

Type of film	Measurement mode	Sensitivity (µA M^−1^)	Stability in 1 mM of H_2_O_2_ (h)
PB	Three-electrode setup	8.8 ± 0.5	0.25
PB-NiHCF	Three-electrode setup	1.6 ± 0.1	4
PB	Short-circuit mode	8.9 ± 0.4	0.25
PB-NiHCF	Short-circuit mode	1.6 ± 0.1	4
**PB/Pt-B**	**Short-circuit mode**	**29 ± 2**	**3.4**

### SECM in short-circuit mode

Visualizing H_2_O_2_ evolution using SECM offers valuable insight into local catalytic activity, reaction kinetics, and spatial distribution of reactive species, making it a powerful tool for studying, e.g., light-driven or electrocatalytic processes [16, 17, 40, 41]. PB-based microsensors operating in a conventional three-electrode setup have been already employed for SECM mapping of H_2_O_2_ generation [26, 27, 42]. Here, we introduce for the first time a short-circuit mode of PB-modified MEs for SECM experiments. In this case, the setup is significantly simplified as an ammeter that can be used with a single combined microsensor (Fig. S7a). Since the microsensor operates without an externally applied potential, there is no risk of sensor damage if the electrode is momentarily exposed to air (e.g., due to cell leakage or evaporation). This enhances operational safety and simplifies handling, particularly during long-term measurements. Additionally, the use of a single combined working/reference electrode minimizes alignment issues and electrode interference in confined electrochemical cells.

**Fig. 7:**
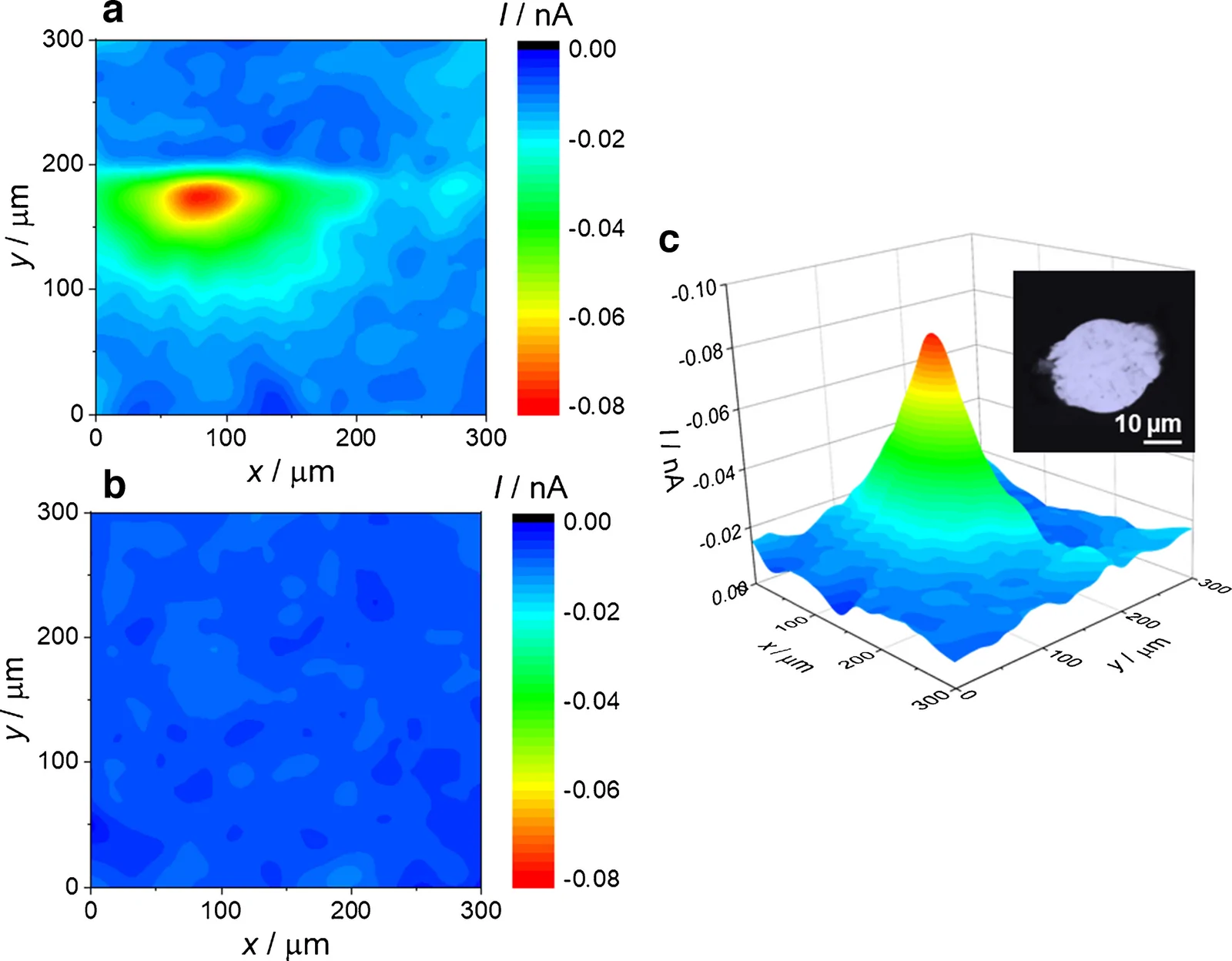
SECM image of an H_2_O_2_-generating Pt wire (shown in inset, Ø = 25 µm): **a** 2D plots of images recorded when the Pt wire was biased at −0.5 V and **b** not biased, and **c** a 3D plot of (**a**); 50 mM K_2_HPO_4_/KH_2_PO_4_, pH 6.0, with 0.1 M KCl; translation rate = 5 μm·s^−1^

As a proof-of-principle measurement, the electrocatalytic H_2_O_2_ evolution was visualized at a planar Au electrode biased at −0.4 V (Fig. S8) and at a Pt ME biased at −0.5 V (Fig. 7) in SECM substrate-generation-tip-collection mode (Fig. S7b). A significant increase in reduction current was observed as the PB-NiHCF-modified Pt-ME approached the H_2_O_2_-generating Au structure (Fig. S8b) or the embedded Pt wire (Fig. 7a,c). Control experiments, performed without applying potential to the substrates, showed no distinguishable signal (Fig. 7b, Fig. S8c). Post-imaging cyclic voltammetry (after 3 h of measurements) in 0.1 M HCl/KCl confirmed the high stability and integrity of the microsensor (Fig. S9).

### ATP release detection at alveolar type II cells (ATII)

ATP is known to be one of the ubiquitous extracellular signaling molecules, which regulate numerous cellular processes through purinergic signaling pathways. Typically, ATP is released via exocytosis or through various channels such as connexin or pannexin hemichannels, maxi-anion channels, or CALHM1 ion channels [43, 44]. Its local detection and the dynamics of ATP release in close spatial proximity to cells are of particular importance, as it allows to address signaling pathways and with that regulation of important cellular functions. ATP concentrations in bulk solution are typically quite low due to dilution (usually nanomolar range). Given the complex matrices for ATP measurements, conventional electrochemical detection using, e.g., Pt transducer to detect H_2_O_2_, the byproduct of enzymatic recognition, relies on relatively high oxidation potentials (0.6–0.7 V vs. Ag/AgCl). These may result in interference from electroactive species commonly present in biological systems. This limitation can be overcome by employing PB as the H_2_O_2_ reduction electrocatalyst.

**Fig. 8:**
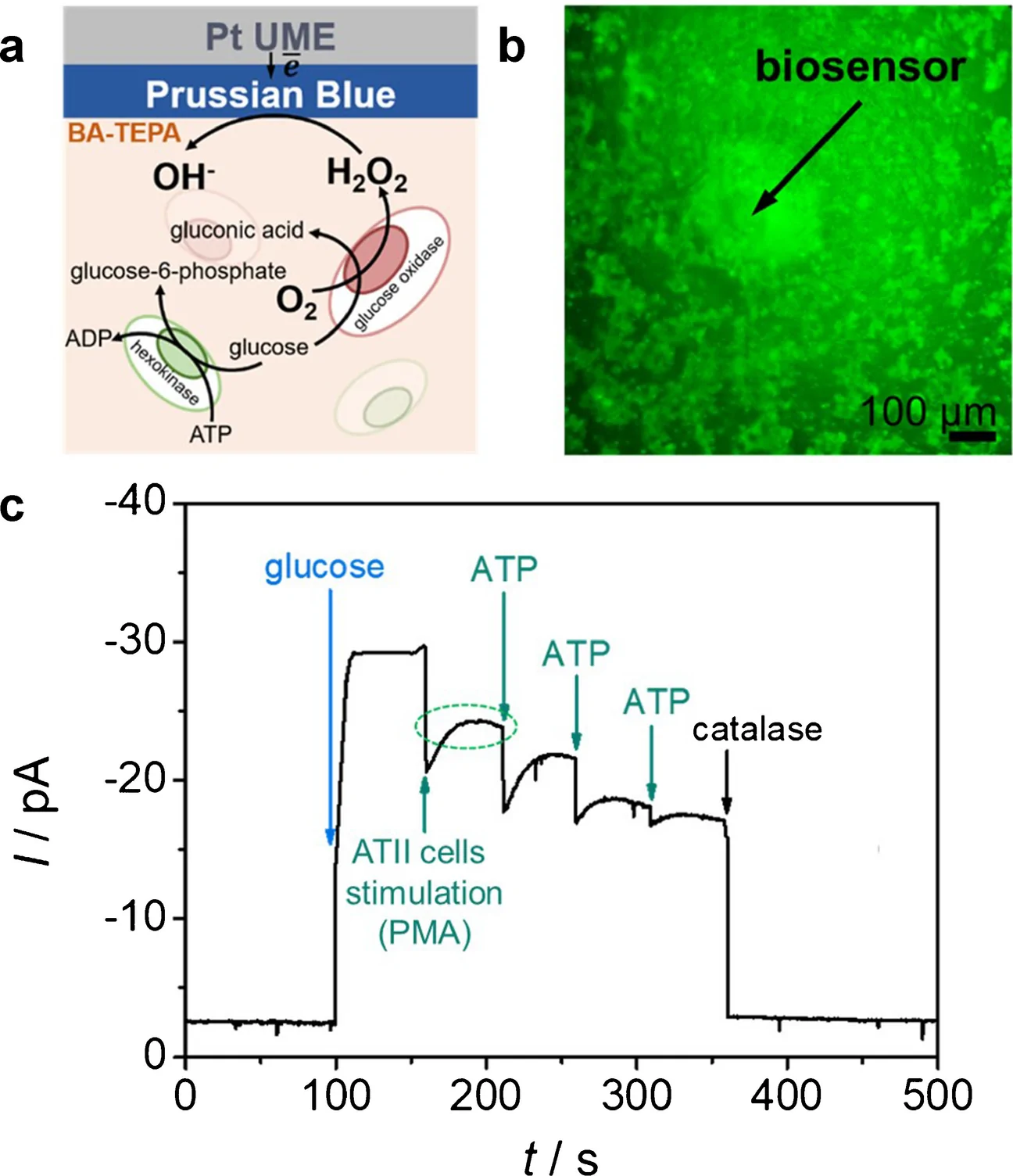
PB-based microbiosensor for ATP detection: **a** scheme of the biosensor operation; **b** optical image of the GFP-labeled ATII cells with the microbiosensor positioned above them; **c** current response of the ATP microbiosensor positioned at a distance of ~ 20 µm at the ATII cells; short-circuit mode; 10 mM Na_2_HPO_4_, 1.8 mM KH_2_PO_4_, 140 mM NaCl, 2.7 mM KCl, 1 mM CaCl_2_, 0.5 mM MgCl_2_, pH 7.4 (Dulbecco’s phosphate-buffered saline)

Figure 8a displays the scheme of the ATP microbiosensor based on PB, which was deposited onto the Pt-ME. Electrochemically induced cross-linking of water-soluble poly(benzoxazine) precursors enables immobilization of GOx and HEX on the PB-modified Pt-ME surface. ATP determination is based on the competitive enzymatic reactions catalyzed by GOx and HEX with the substrate glucose [45]. The electrochemical signal generated by the reduction of H_2_O_2_ (a byproduct of glucose oxidation) is decreasing in the presence of ATP as glucose is consumed in the HEX-catalyzed reaction (Fig. S10a). This change in cathodic current is proportional to the ATP concentration. The sensitivity was calculated from the initial slope of the calibration graph (Fig. S10b).

Both the amount of electrocatalyst (PB) and the duration of the enzyme immobilization procedure were evaluated to maximize the sensitivity of the microbiosensor (Fig. S11). The fabricated biosensors (as single microsensor) were tested in short-circuit mode. The sensitivity increases fourfold with the number of PB deposition cycles, reaching a maximum at 4 cycles, followed by a gradual decrease at higher cycle numbers. A similar trend with a maximum at 14 pulses is observed for the dependence of sensitivity on the number of BA-TEPA deposition pulses. The obtained PB-based microbiosensor possesses 2 times higher sensitivity (91 ± 3 pA µM^−1^, LOD of 9.9 ± 0.3 nM) compared to using a Pt-ME as transducer (48 ± 5 pA µM^−1^) [35]. The obtained linear range of 0.15–2 µM ATP is reduced in comparison to Pt-based microbiosensor (0.3–4 µM). However, the dynamic range of the PB-based biosensor is extended up to 20 µM ATP compared to 16 µM for Pt-based one, which makes the PB-based microbiosensors employable for bioanalytical applications [35]. Another advantage of the PB-based microsensors is their storage stability. ATP microbiosensors based on PB can be stored dried in the refrigerator (+ 4 °C) for at least 1 month without loss of sensitivity (Fig. S12).

The elaborated microbiosensor (“single-electrode” configuration) was applied for ATP release detection at ATII cells. An inverted fluorescence microscope was used to approach the ME in proximity (~20 µm) to the cell-layer attached to the bottom of a Petri dish (Fig. 8b). After a stable baseline was obtained, 10 mM glucose was added to the solution, and the cells were stimulated with 300 nM PMA to elicit exocytosis of ATP-containing lamellar bodies [46, 47]. A change in cathodic current of 5 pA was observed, indicating extracellular ATP release (Fig. 8c). As a control, the same experiment was performed at an area without ATII cells (blank Petri dish). After an initial sharp decrease in the current response, which is related to mechanical disturbance by adding PMA to solution, the signal slowly increased and returned to its initial value after about 2 min (Fig. S13).

To demonstrate that the saturation of the current response-concentration dependence was not reached and to estimate the level of extracellular ATP, three consecutive 0.7 µM ATP additions were made. For three different samples, the average concentration of released ATP was 2.0 ± 0.5 µM, consistent with previously reported values (ATP concentrations in the low micromolar range have been reported [48]). To validate that the signal change was associated with H_2_O_2_ related to the enzymatic conversion of ATP, catalase (1 mg mL^−1^), an enzyme that decomposes H_2_O_2_, was added to the solution. This resulted in a decrease in cathodic current to the baseline level (within 5% deviation) (Fig. 8c).

## Conclusion

In this study, we show the development of advanced electrochemical H_2_O_2_ microsensors based on PB and PB-NiHCF operated in short-circuit mode. The microsensors exhibit high selectivity, e.g., only more than a 10-fold excess of ascorbate affects the sensor signal to micromolar levels of H_2_O_2_, which is crucial for measurements in biological systems. Both sensitivity and operational stability of the PB- and PB-NiHCF-based microsensors in short-circuit mode remain unchanged compared to the conventional three-electrode setup. Incorporation of a porous Pt-B sublayer results in a threefold increase in sensitivity. Moreover, a high operational stability was observed for the PB/Pt-B-modified MEs. The current response retains the initial value for 3.4 h in 1 mM H_2_O_2_ solution. The developed micro(bio)sensors were successfully applied for SECM mapping of H_2_O_2_ evolution and detection of ATP release from stimulated ATII cells. In prospect, the range of analytes can be expanded by immobilizing other enzyme-oxidases onto the PB film. With further miniaturization, these micro(bio)sensors hold promise for local in situ investigations of metabolic activity at the intracellular level.

## Supplementary Information

Below is the link to the electronic supplementary material.

Supplementary file1 Cyclic voltammograms of PB and PB-NiHCF films; stability studies of integrated Ag QRE; selectivity tests toward common interfering species; characterization of Pt-black modified electrodes (SEM, cyclic voltammograms, calibration curve); supplementary SECM setup schematics and experimental configuration; SECM maps of H_2_O_2_ generation on a gold substrate; optimization of fabrication procedure, calibration curve and storage stability of the ATP microbiosensor; current responses of the microbiosensor to ATII cells stimulation in the presence and absence of the cells. (DOCX 652 KB)

## Data Availability

Data will be made available on request.
