# Supplementary material for: Prussian Blue–based microsensors in short-circuit mode: a simplified approach for localized hydrogen peroxide detection
Source: Anal Bioanal Chem. 2026 Jun 9;418(23):7981–91. doi: 10.1007/s00216-026-06586-z (PMC13744564; doi:10.1007/s00216-026-06586-z)
Supplement: Supplementary file 1 [file 216_2026_6586_MOESM1_ESM.docx]

**Supplementary Information**

Analytical and Bioanalytical Chemistry

**Prussian Blue-based microsensors in short-circuit mode: a simplified approach for localized hydrogen peroxide detection**

Elena Daboss^a*^, Sven Daboss^a^, Manfred Frick^b^, Christine Kranz^a^

^a^ Institute of Analytical and Bioanalytical Chemistry, Ulm University, Albert-Einstein-Allee 11, 89081 Ulm, Germany

^b^ Institute of General Physiology, Ulm University, Albert-Einstein Allee 11, Ulm 89081, Germany

* Corresponding author. Dr Elena Daboss, email address: [elena.daboss@uni-ulm.de](mailto:elena.daboss@uni-ulm.de)

# Fig. S1 Cyclic voltammograms of Pt-ME modified with PB vs. Ag QRE (dashed line) and external Ag/AgCl reference electrode (solid line), 20 mV s^-1^, 0.1 M KCl in 0.1 M HCl

Fig. S2 Current responses to interfering compounds and H_2_O_2_ of a Pt-ME (0.6 V vs. Ag/AgCl) (grey curve), a Pt-ME modified with Pt-B (0.3 V vs. Ag/AgCl) (black curve) and a Pt-ME modified with PB (short-circuit mode) (blue curve)

Fig. S3 Long-term stability of Ag QRE potential; the electrode was stored in air (**□**) and in PBS with pH 7.4 (**○**)

Fig. S4 Deposition of PB/Pt-B onto Pt-ME and corresponding characterization steps: (a) Pt-B deposition from 31 mM H_2_PtCl_6_ and 0.67 mM Pb(NO_3_)_2_ in PBS, applied potential: –0.06 V vs. Ag/AgCl; (b) cyclic voltammograms of Pt-ME before and after deposition of Pt-B recorded in 10 mM [Ru(NH_3_)_6_]Cl_3_ with 0.1 M KCl, scan rate: 0.1 V s^-1^; (c) PB deposition from 1.25 mM K_3_[Fe(CN)_6_], 1.25 mM FeCl_3_, 0.1 M KCl in 0.1 M HCl, scan rate: 0.02 V s^-1^; (d) cyclic voltammograms of PB/Pt-B/Pt-ME recorded before (blue) and after (teal) three consecutive calibrations in 0.1 M KCl with 0.1 M HCl, scan rate: 0.02 V s^-1^

Fig. S5 Cyclic voltammogram of Pt-B/Pt-ME in 0.5 M H_2_SO_4_, scan rate: 0.2 V·s^-1^. To calculate the EASA of the deposited Pt-B layer, the hydrogen desorption peak (at around –0.2 V) was integrated. The obtained integral was divided by the scan rate and the respective charge of Q = 208 µC cm^-2^

Fig. S6 Current responses of the H_2_O_2_ sensor based on PB/Pt-B recorded in short-circuit mode; 50 mM K_2_HPO_4_/KH_2_PO_4_, pH 6.0, with 0.1 M KCl

Fig. S7 The schemes of (a) the setup used for the SECM measurements and of (b) the sample generation – tip collection mode

Fig. S8 (a) Optical image of a H_2_O_2_-generating gold structure, mapped area marked red; 2D-SECM images of the gold structure recorded when (b) the gold was biased at ‒0.4 V and (c) not biased; translation rate = 5 μm·s^-1^

Fig. S9 Cyclic voltammograms of PB-NiHCF-based microsensor recorded before (blue) and after (teal) SECM mapping; 0.1 M KCl with 0.1 M HCl, scan rate: 0.02 V s^-1^

Fig. S10 (a) Current responses of the ATP biosensor based on PB recorded in short-circuit mode and (b) corresponding calibration graph (n = 3); 10 mM Na_2_HPO_4_, 1.8 mM KH_2_PO_4_, 140 mM NaCl, 2.7 mM KCl, 1 mM CaCl_2_, 0.5 mM MgCl_2_, pH 7.4 (Dulbecco's PBS)

Fig. S11 Sensitivity of ATP microbiosensors based on PB (n=3) as a function of the number of PB deposition cycles and a function of the number of BA-TEPA deposition pulses. The microbiosensors were evaluated in short-circuit mode; 10 mM Na_2_HPO_4_, 1.8 mM KH_2_PO_4_, 140 mM NaCl, 2.7 mM KCl, 1 mM CaCl_2_, 0.5 mM MgCl_2_, pH 7.4 (Dulbecco's PBS). Solid and dashed lines show sensitivity and standard deviation for the microbiosensors based on the same enzyme immobilization protocol without a PB layer ^[1]^

Fig. S12 Storage stability of ATP microbiosensors based on PB (n = 3); biosensors were stored dried at +4 °C

Fig. S13 Current responses of the ATP microbiosensor to PMA addition in the presence and absence of the ATII cells; short-circuit mode; 10 mM Na_2_HPO_4_, 1.8 mM KH_2_PO_4_, 140 mM NaCl, 2.7 mM KCl, 1 mM CaCl_2_, 0.5 mM MgCl_2_, pH 7.4 (Dulbecco's PBS)

1. Ziller C, Lin J, Knittel P, Friedrich L, Andronescu C, Pöller S, et al. Poly(benzoxazine) as an Immobilization Matrix for Miniaturized ATP and Glucose Biosensors. ChemElectroChem. 2017;4(4):864–71. https://doi.org/10.1002/celc.201600765
